# Growth-predation risk tradeoffs constrain the local distribution of a thicket-forming Staghorn coral to marginal reef habitats

**DOI:** 10.1038/s41598-025-21028-z

**Published:** 2025-10-23

**Authors:** Mark C. Ladd, Thomas C. Adam, Dana T. Cook, Deron E. Burkepile, Andrew J. Brooks, Russell J. Schmitt, Sally J. Holbrook

**Affiliations:** 1 Department of Ecology, Evolution and Marine Biology, University of California Santa Barbara, Santa Barbara, CA 93106 USA; 2 Marine Science Institute, University of California Santa Barbara, Santa Barbara, CA 93106 USA; 3 Present Address: Population and Ecosystems Monitoring Division , NOAA Southeast Fisheries Science Center , Miami, FL USA

**Keywords:** Coral predation, Corallivory, Foundation species, Acropora, Ecological tradeoff, Moorea, Community ecology, Restoration ecology

## Abstract

On tropical reefs, environmental conditions and biological interactions are fundamental drivers of the spatial distribution of corals, which in turn influences community attributes and ecosystem rate processes. Here we focus on a major habitat-providing species of coral, the thicket-forming staghorn *Acropora pulchra*, in Moorea, French Polynesia, to explore environmental attributes that shape its local distribution. At the island scale, *A. pulchra* decreased in abundance with increasing distance from the shoreline and was inversely related to nitrogen enrichment. To investigate growth-predation risk tradeoffs, we quantified growth and corallivory rates across major abiotic (nutrients, sedimentation) and biotic (corallivorous fish biomass) gradients. At 20 sites divided between fringing reef and mid-lagoon habitats, we quantified colony growth of transplanted *A. pulchra* fragments (nubbins) that were either exposed or protected from predators after 83 days. Nubbins protected from corallivores grew more in the mid-lagoon than on the fringing reef, whereas mid-lagoon nubbins exposed to predators only achieved 30% of the growth of exposed fringing reef nubbins. These findings suggest that a growth-predation risk tradeoff exists for *A. pulchra*, with predation a major constraint on the local distribution of *A. pulchra* thickets that hinders the ability of staghorn to proliferate further offshore from the fringing reef.

**Supplementary Information:**

The online version contains supplementary material available at 10.1038/s41598-025-21028-z.

## Introduction

Coral reefs are among the most threatened ecosystems on Earth, highlighting the need to understand environmental attributes that constrain their local distribution and abundance. Corals are particularly susceptible to nutrient pollution and sedimentation^1–4^, and the distribution and intensity of these land-based stressors are often heterogeneous across a seascape^5,6^. These and other environmental attributes typically create a mosaic of local conditions that, from the perspective of coral physiology, range from optimal to marginal to uninhabitable. Nutrient pollution can exacerbate the intensity and duration of coral bleaching from marine heat waves^7^, impair coral growth^8^, increase the likelihood and severity of coral disease^9^, and can favor fast-growing benthic competitors like seaweeds^10^, which can result in a persistent regime shift^11–13^. Similarly, increased sediment loads can cause partial or complete mortality of corals^14^, decrease growth via increased water turbidity^15–17^, and increase the prevalence of coral disease^18^. In addition to these pervasive human stressors, reef-building corals can be limited by their predators. Predation on corals (i.e., corallivory) is often taxon-specific^19–22^ and varies substantially within^21^ and among habitats^23^, generating a heterogenous landscape of predation. Coral predation can limit the local habitat distribution of some coral species, such as the coral *Madracis marabilis* in the Florida Keys, USA^24^ and *Pocillipora* spp. and *Pavona gigantea* on Pacific reefs of Panama^25^. Moreover, predation can increase coral susceptibility to pervasive stressors such as thermal stress^26,27^, amplifying the impacts of coral predation. Consequently, the impacts of consumers could force foundation species such as corals to exist in physiologically suboptimal habitats where they perform less well but are able to survive.

As environmental conditions continue to degrade on tropical reefs across the globe^28^, it is critical to understand the abiotic and biotic mechanisms that shape local patterns of habitat distribution and the abundance of key species. Such information is fundamental to predicting future dynamics under changing conditions, managing for resilience, and developing options for restoration^29,30^. However, this information is often lacking, particularly for important habitat-forming foundation species^30^. Here, we assessed the role of several key abiotic and biotic forces in shaping the local distribution and abundance of a major habitat-provisioning coral species, *Acropora pulchra*^31,32^. We surveyed 188 sites in the shallow lagoons surrounding the island of Moorea, French Polynesia to examine the relationship between the distribution and abundance of *A. pulchra*, distance to shore, and nutrient pollution. We then conducted a series of field experiments to test two hypotheses that could give rise to the observed spatial patterns of the distribution of this foundation species around Moorea. Field experiments quantified coral growth and corallivory across varying levels of abiotic and biotic factors to test these hypotheses. The first hypothesis is that abiotic conditions promote faster growth of *A. pulchra* at nearshore habitats (i.e., fringing reefs) compared to offshore (i.e., mid-lagoon) habitats. The second hypothesis is that when exposed to corallivorous fishes, *A. pulchra* fares worse in the mid-lagoon compared to the fringing reef due to higher rates of predation.

## Results

**Fig. 1:**
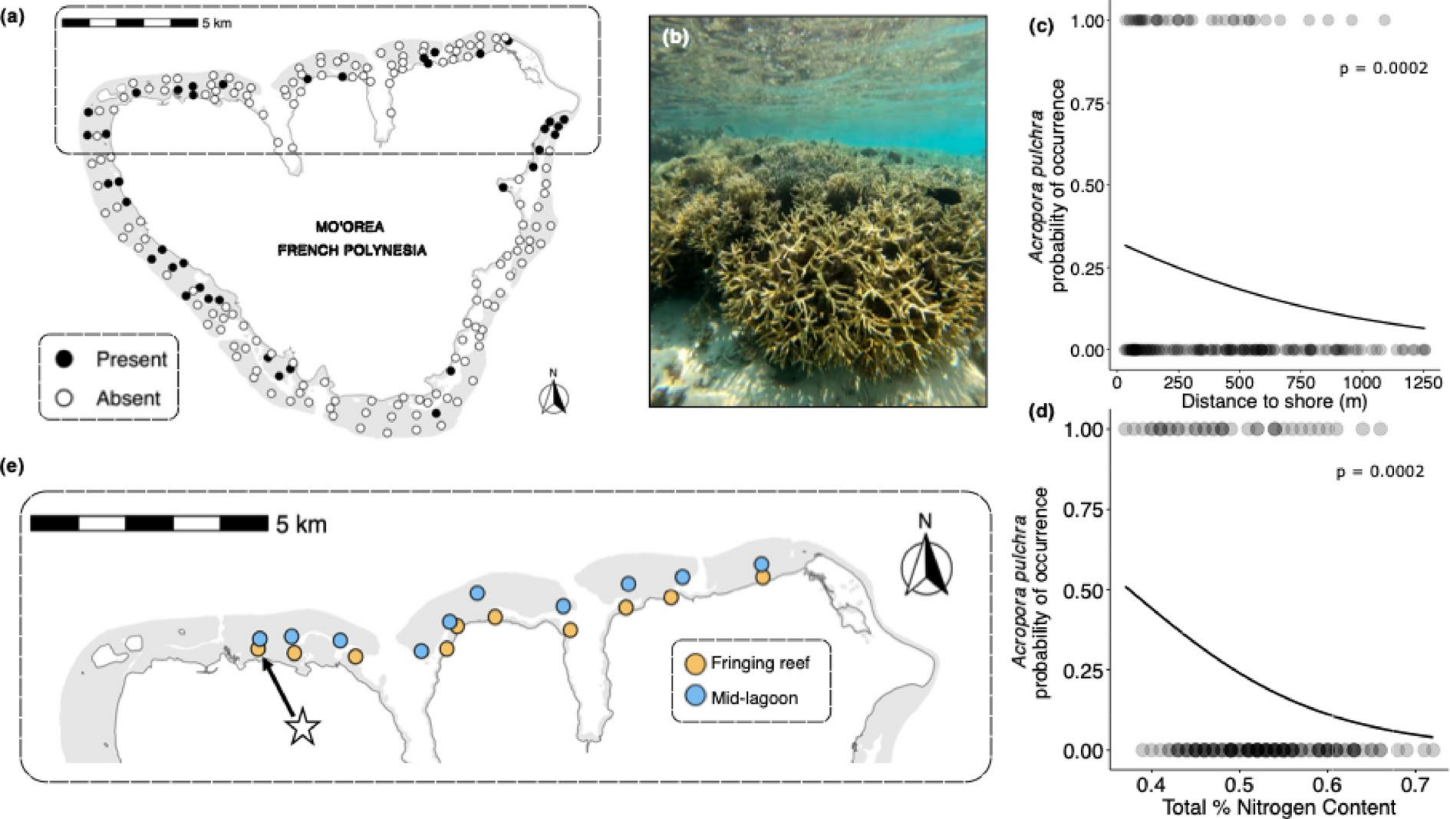
(**a**) Map of 188 sites sampled in 2016 on the three shores of Moorea, French Polynesia. Black dots represent sites where large (> 1 m diameter) stands of *A. pulchra* were present, white dots represent sites where *A. pulchra* was absent. (**b**) In-water photograph of *A. pulchra* stands on the north shore of Moorea (photo credit: C. Clements). (**c**) Presence-absence of *Acropora pulchra* with increasing distance from the shoreline and (**d**) Presence-absence of *Acropora pulchra* with %N derived from *Turbinaria ornata* tissue samples. Data for (**c**) and (**d**) from surveys conducted at 188 sites on all shores of Moorea. Statistics from generalized linear models. (**e**) Study sites on the north shore of Moorea, French Polynesia where the field experiment was conducted. Yellow points indicate fringing reef sites (*n* = 10), blue points indicate mid-lagoon sites (*n* = 10). Star denotes location where experimental corals were collected.

With respect to presence or absence of *A*. *pulchra*, large thickets (≥ 1 m diameter) were encountered at 39 of the 188 sites in the island-wide surveys (Fig. 1a). The presence of *A*. *pulchra* thickets (Fig. 1b) was negatively related to distance to shore (GLM: χ^2^(1) = 13.65, *p* = 0.0002; Fig. 1c) and nutrient enrichment (as estimated by total %N in tissue of the macroalga *Turbinaria ornata*) (GLM: χ^2^(1) = 14.26, *p* = 0.0002; Fig. 1d) with no interaction between the two factors (GLM: χ^2^(1) = 0.01, *p* = 0.92) and no effect of island shore (GLM: χ^2^(1) = 5.16, *p* = 0.08). There was a significant negative correlation between Total %N and distance to shore (Pearson’s correlation: *p* = 0.001; *r* = −0.25) indicating that N availability decreased with increasing distance from the shoreline.

**Fig. 2:**
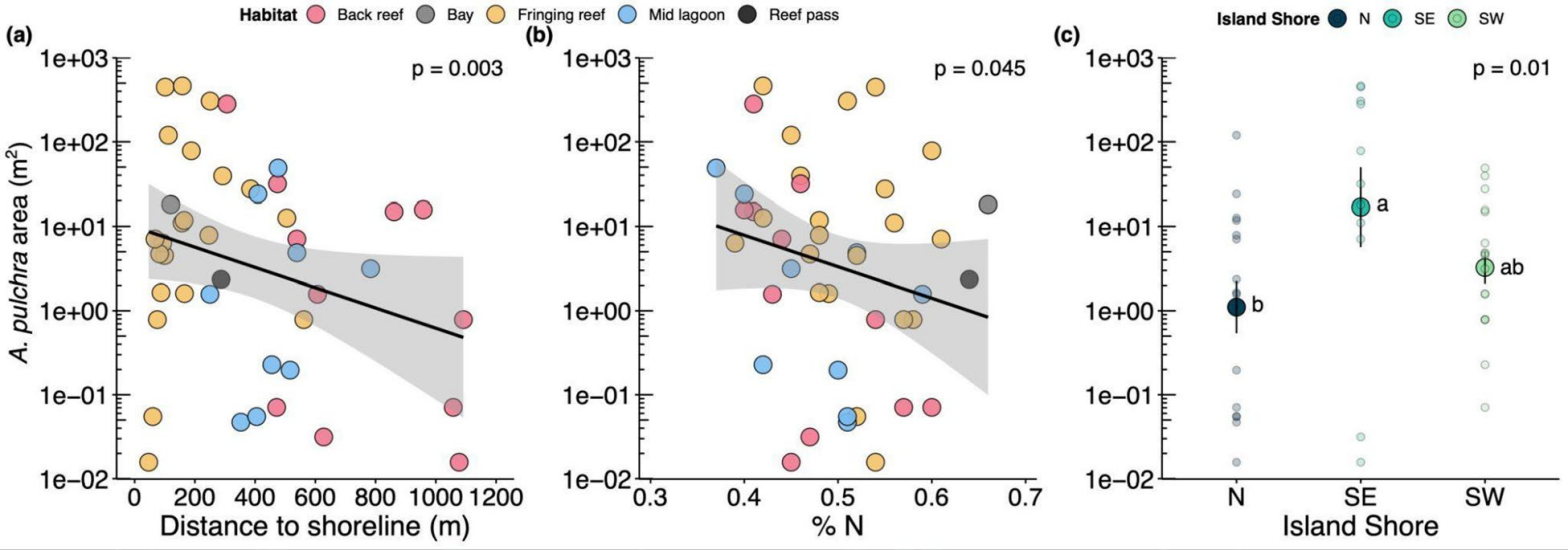
Area (m^2^) of *Acropora pulchra* observed during island-wide surveys regressed against (**a**) distance to shoreline (m) and (**b**) % N tissue content of *Turbinaria ornata* collected from each site. (**c**) Mean area (m^2^) ± SE of *A. pulchra* observed on each island shore (large solid points). Individual points represent raw data; black lines for (**a**) and (**b**) are fitted linear model trendline with 95% confidence intervals (gray shading). For (**c**), points that share a letter are not statistically different per *post hoc* analyses. Data shown are from the 43 sites where *A. pulchra* (> 10 cm) was observed during surveys conducted in May of 2016. Statistics are from linear mixed effects models.

In addition to spatial heterogeneity in the prevalence (presence or absence) of thickets, spatial patterns in the abundance (i.e., planar area) of thickets emerged from our analyses. The total abundance of *A. pulchra* at a site was inversely related to distance from shore (GLM: χ^2^(1) = 8.90, *p* = 0.003; Fig. 2a), %N (GLM: χ^2^(1) = 4.02; *p* = 0.045; Fig. 2b), and varied by island shore (GLM: χ^2^(2) = 8.96; *p* = 0.01; Fig. 2c), with the southeast shore having significantly more *A. pulchra* compared to the north shore (*post hoc* test with Tukey’s correction: *p* = 0.014). The majority (77%) of the total abundance (planar area) of *A. pulchra* recorded was found in the fringing reef habitat, with 74% of all *A. pulchra* area recorded in the island-wide surveys located at sites within 250 m of the shoreline.

**Fig. 3:**
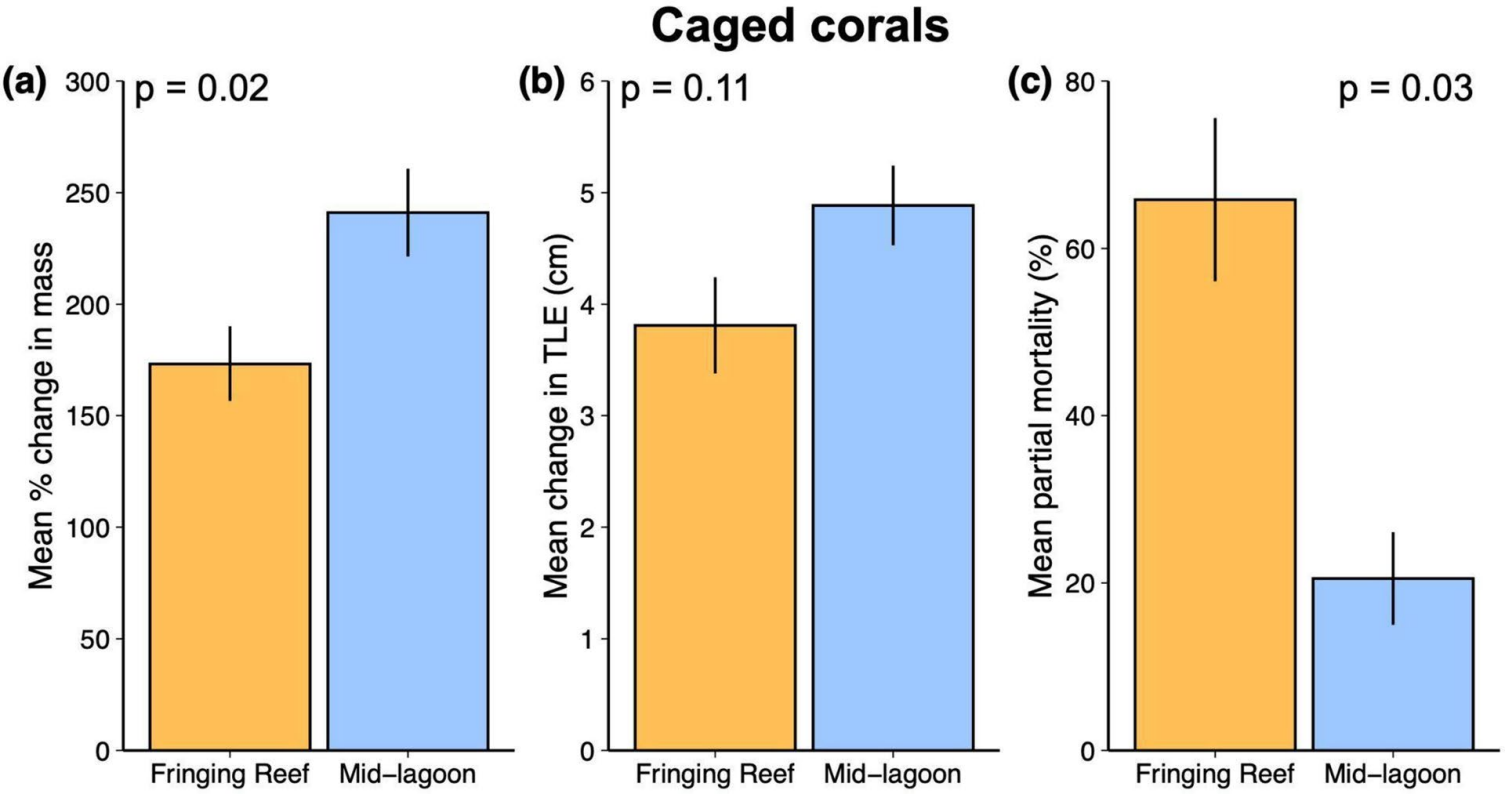
(**a**) Mean percent change in mass, (**b**) total linear extension (TLE; cm) and (**c**) partial mortality for experimental corals growing in the absence of predation (i.e., caged treatments only; *n* = 99) on the fringing reef and mid-lagoon. Only corals that displayed some level of partial mortality (i.e., > 0) are included in plot (**c**). Values are means ± SE. Statistics are from linear mixed effects models.

Regarding our first hypothesis that the fringing reef habitat is more favorable for coral growth, we found that the mean percent change in the mass of corals deployed in cages and thus protected from predation was 1.4x higher in the mid-lagoon compared to the fringing reef (Cohen’s d = 0.53; GLMM: estimate = 2.03, SE = 0.87, z = 2.33, *p* = 0.02; Fig. 3a). The estimated Cohen’s d value indicates an effect size between medium (0.5) and large (0.8) of habitat on growth in the absence of corallivorous fishes. We did not detect a difference in the change in total linear extension (TLE) of caged corals deployed in the two habitats (Cohen’s d = 0.61; GLMM: estimate = 1.08, SE = 0.68, z = 1.59, *p* = 0.11; Fig. 3b). The hurdle model revealed that the likelihood of observing any coral mortality did not differ between habitats (binomial GLMM: estimate = −0.47, SE = 0., z =−2.2, *p* = 0.35). However, when we assessed only corals that displayed some level of partial mortality, we found that partial mortality was more than 3x higher for caged corals deployed to the fringing reef compared to those deployed to the mid-lagoon (Cohen’s d = 1.32; GLMM: estimate = −1.67, SE = 0.76, z = −2.2, *p* = 0.03; Fig. 3c).

**Fig. 4:**
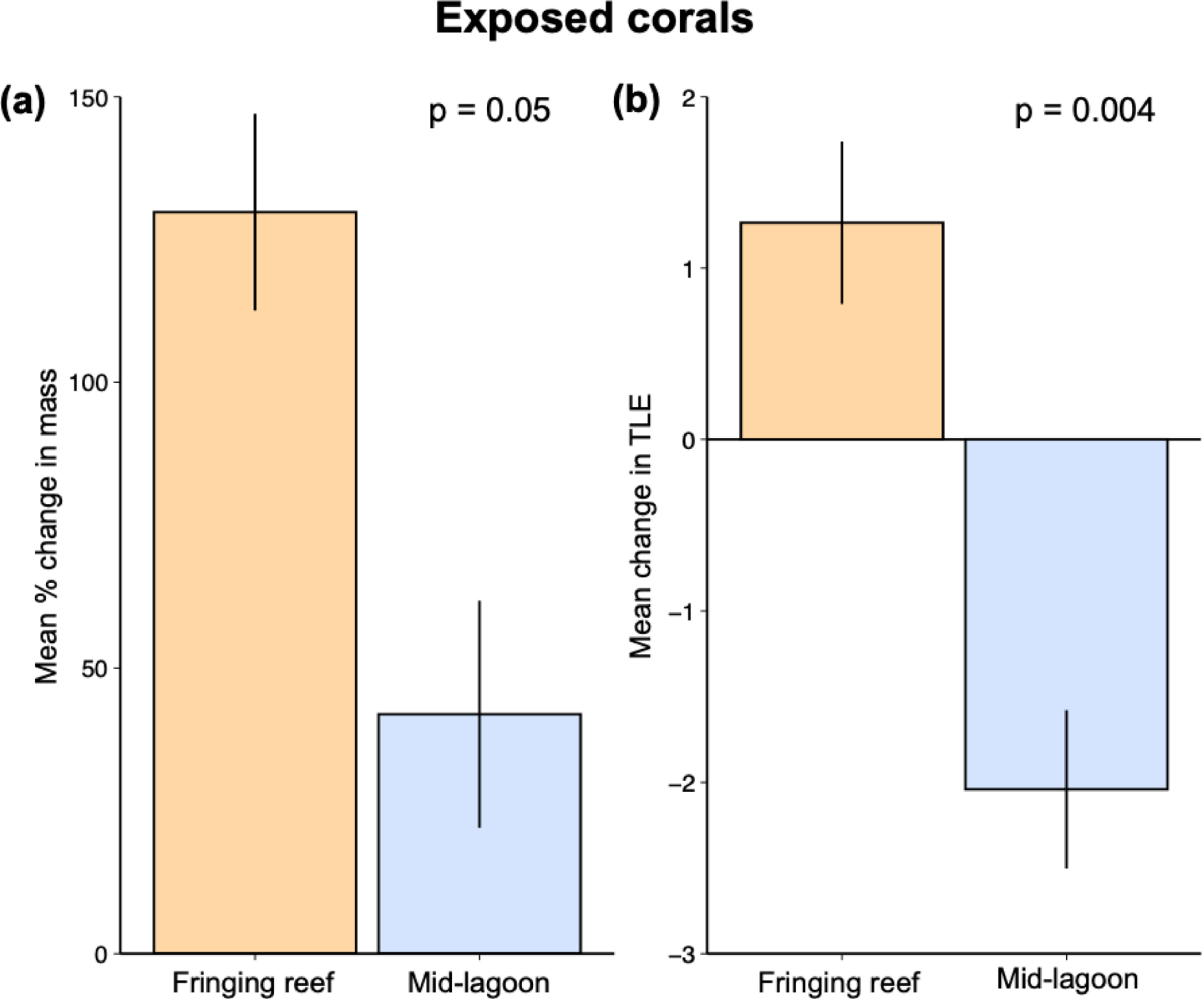
(**a**) Mean percent change in mass, and (**b**) total linear extension (TLE; cm) for experimental corals growing in the presence of predation (i.e., exposed treatments; *n* = 100) on the fringing reef and mid-lagoon.

**Fig. 5:**
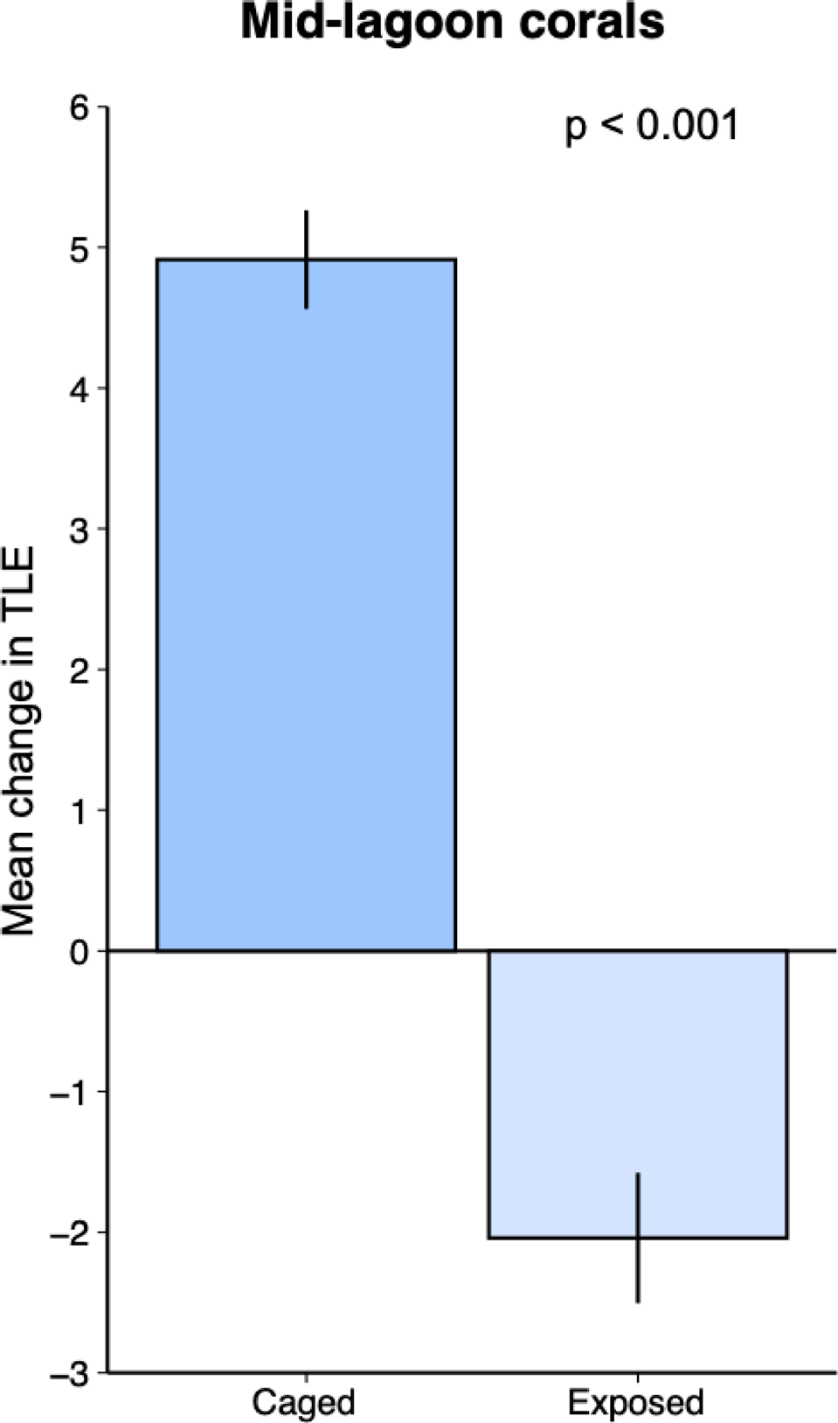
Mean total linear extension of caged and exposed experimental corals transplanted to the mid-lagoon (*n* = 100). Values are means ± SE. Statistics are from linear mixed effects models.

Our second hypothesis that *A. pulchra* performs worse when exposed to corallivorous fishes in the mid-lagoon compared to the fringing reef was supported by our findings. In contrast to corals protected by cages (Figure S1), exposed corals deployed to the fringing reef increased 3x more in mass than exposed corals in the mid-lagoon (mean percent change in mass ± SD; 130% ± 17 vs. 42% ± 20) (Fig. 4a). The estimated Cohen’s d value (0.69) indicates a median effect size of habitat type on nubbin growth in the presence of corallivorous fishes, although this difference was only marginally significant, likely to due to high variability in growth rates (GLMM: estimate = −90.7, SE = 46.6, z = −1.97, *p* = 0.052). There was, however, a significant effect of habitat on our second growth metric, total linear extension (TLE; Cohen’s d = 0.96; GLMM: estimate = −3.22, SE = 1.13, z = −2.84, *p* = 0.004; Fig. 4b), which indicated a large effect size of habitat type. Exposed corals on the fringing reef added on average 1.26 cm ± 0.47 TLE over the course of the experiment, compared to exposed corals on the mid-lagoon that on average lost 2.04 cm ± 0.46 TLE. When we compared change in TLE for mid-lagoon corals only, we found that corals protected from predators (i.e., caged) increased in TLE nearly 7 cm more than those exposed to predators (Cohen’s d = 2.32; GLMM: estimate = −1.36, SE = 0.10, z = −13.24, *p* < 0.001; Fig. 5). The high (2.32) Cohen’s d value indicates a very large effect size of caging on change in TLE in the mid-lagoon habitat. This also suggests that the net reduction in TLE for exposed corals in the mid-lagoon was not due to hydrodynamic forces breaking off pieces of coral.

The estimated mean biomass (g m^−2^) of corallivorous fishes (Table S1) was similar at mid-lagoon sites compared to the fringing reef sites (mean ± SE; 21.48 g m^−2^ ± 3.59 vs. 20.32 g m^−2^ ± 3.91, respectively), with no difference in the overall mean biomass detected between habitats (Habitat effect: F_1,18_ = 0.13; *p* = 0.80; Figure S2a). In contrast to overall mean corallivore biomass, we found that corallivorous fish community composition based on biomass differed significantly between the fringing reef and mid-lagoon (PERMANOVA: F_1,18_ = 1.26, R^2^ = 0.12, *p* = 0.038; Figure S3a). Two species of parrotfishes, *Chlororus spilurus* and *Scarus psittacus*, had a higher biomass in the mid-lagoon compared to the fringing reef and were the two main species contributing to observed dissimilarity between habitats, contributing to 38.6% and 32.5% of the observed differences, respectively (Table S2). We did not detect any difference in corallivorous fish community composition based on abundance (PERMANOVA: F_1,18_ = 1.26, R^2^ = 0.07, *p* = 0.29; Figure S3b). There was no relationship between mean corallivorous fish biomass and coral performance (% growth caged - % growth exposed; F_1,18_ = 0.51; *p* = 0.48; Figure S2d). Mean nitrogen tissue content of *Turbinaria ornata* collected in August of 2017 at each experimental site ranged from a low of 0.31% to a high of 0.58% (mean ± SE = 0.48 ± 0.016%), but did not differ by habitat (F_1,18_ = 0.008; *p* = 0.93; Figure S2b). Although sedimentation rates varied 4-fold among sites, the difference between habitats was just at the margin of statistical significance with the trend for greater sedimentation on fringing reefs (F_1,18_ = 3.96; *p* = 0.06; Figure S2c). We found no effect of nitrogen tissue content (F_1,18_ = 0.93; *p* = 0.35; Figure S2e) or sedimentation rates (F_1,18_ = 0.21, *p* = 0.65; Figure S2f) on the performance of the coral nubbins (i.e., finger-like fragments; Figure S1) that were protected from corallivorous fishes.

## Discussion

Because foundation species have a disproportionate influence on ecosystem properties, knowledge of how habitat-specific attributes influence their distribution and abundance is essential for predictive insight on ecosystem responses to local and global stressors^33^. In turn, such understanding is crucial for guiding effective management and conservation practices. On coral reefs, habitat-providing coral such as branching acroporids that form extensive thickets are among the most important foundation species due to their profound contributions to local and regional biodiversity, influence on community structure and dynamics, and modulating effect on key ecosystem processes^32^. Habitat differences significantly influence the growth of corals and their risk of predation. Here, we found that for the thicket-forming staghorn coral *Acropora pulchra*, predator-driven losses in biomass overwhelmed the positive effect of abiotic conditions that supported higher coral growth rates and lower partial mortality in the mid-lagoon compared to the fringing reef habitat. This apparent ecological tradeoff between growth and predation risk fosters a marked cross-reef distribution of staghorn thickets in Moorea, which primarily are located within several hundred meters of the shore in the lagoons that encircle the island. We found small *A. pulchra* individuals were distributed across the entire span of the lagoon - from the fringing reef to the back reef behind the barrier reef crest - which supports the notion that the highly spatially skewed distribution of staghorn thickets arises in large part from post-recruitment processes. Our experiments suggest that the emergent cross-lagoon distribution of *A. pulchra* thickets reflects habitat differences in predation intensity, despite the potential for much faster rates of coral growth in riskier lagoon habitats.

Growth–predation risk tradeoffs have been invoked to explain other striking cross-habitat distributions of coral reef organisms. For example, Grol et al.^34^ found that habitat segregation between juvenile and adult French grunts (*Haemulon flavolineatum*) in the Caribbean, where adults inhabit the reef and juveniles occupy mangrove and seagrass habitats, was driven primarily by an extreme risk of predation on young grunts in coral habitats. Similar to our finding of faster *Acropora* growth in the riskier habitat, growth rates of juvenile grunts were greater on the highly dangerous reef than in safer mangrove or seagrass habitats^34^. Tradeoffs between maximizing growth and minimizing predation risk reflect a fundamental ecological tension for prey between the need to acquire resources and the need to avoid being killed by a predator. There is a suite of behavioral mechanisms that mobile organisms can use to balance risk and reward, such as reef fishes or terrestrial antelope weighing up food reward and perceived risk when making habitat- or patch-level foraging decisions^35,36^. By contrast, sessile species like corals or plants rely heavily on passive strategies, such as developing structural defenses, chemical deterrents^37,38^, or occupying locations with fewer predators^39^. This difference implies that mobile species can respond dynamically to changing conditions, while sessile species depend more on long-term adaptations to their local environment to mediate growth-predation tradeoffs.

On tropical reefs, coral predation (corallivory) can exert substantial pressure on coral populations and influence the local distribution of preferred prey species^19,24,40–42^. Corallivory often varies among coral reef habitats^23,43,44^ and along abiotic gradients that can also influence coral success like depth^45^ or hydrodynamic forces^21^. In this study, experimental *A. pulchra* colonies that were exposed to coral predators and transplanted to the fringing reef had growth rates nearly 3x higher than those transplanted and exposed to predators in the mid-lagoon. These patterns in growth were the opposite of our results for caged corals that were protected from predators, indicating a tradeoff between predation and growth conditions in the mid-lagoon and fringing reef habitats. However, our fish surveys did not detect a difference in the biomass of coral predators between habitats, precluding our ability to link our observed predation effect to predator abundance. This surprising result was likely due to the ability for relatively cryptic and rare corallivorous fish species, such as pufferfish and triggerfish^46^, to drive patterns in predation between habitats. Similar to our findings, previous experimental work using *A. pulchra* at two sites in the mid-lagoon of the north shore of Moorea reported the near-complete removal (64% to 100%) of colonies similar in size to those used in our experiment within one week and one month of deployment^1,47^ respectively). Other studies conducted in the lagoons of Moorea using congeners of *A. pulchra* have also reported high rates of predation on juvenile-sized fragments^39^. While the impacts of coral predation on individual colonies can range from benign to lethal^20,23^, smaller corals are more susceptible to complete mortality compared to larger corals^20,48^. For example, incidental predation on newly recruited corals can drive mortality and contribute to early life history stage bottlenecks, ultimately shaping the distribution of corals across the seascape^49^. Thus, predation is particularly relevant for recruits and juvenile corals as complete removal by predators could serve as a mechanism impeding the recovery of coral populations in the wake of a coral-killing disturbance.

Predation as a mechanism influencing the distribution of *A. pulchra* in the mid-lagoon is most likely to influence small juvenile corals rather than larger extant thickets. *Acropora pulchra* are tightly associated with the damselfish *Stegastes nigricans* and *S. punctatus* on reefs throughout the Pacific^31^. *Stegastes* spp. are aggressive, highly territorial fish that fiercely protect their algal turf gardens from intruders^32,50–53^. However, *Stegastes* spp. may be less likely to colonize *A. pulchra* until the coral colony has reached sufficient size^54^, leaving a window during which these young colonies are particularly vulnerable to predation. Thus, while it is unlikely that predation influences the persistence of large, *Stegastes*-defended thickets of *A. pulchra*, it may exert a strong influence on the ability for *A. pulchra* to re-establish in areas of high predation intensity. Consequently, biological interactions may be especially important in shaping the distribution of *A. pulchra* around the island of Moorea whereby predation largely restricts *A. pulchra* to the marginal fringing reef habitats, while protection of *A. pulchra* colonized by damselfishes allows them to persist in the favorable mid-lagoon habitat where they are able to grow more rapidly. Although disentangling the role of *Stegastes* in driving patterns of the distribution of *A. pulchra* was beyond the scope of this study, understanding the role of these ecosystem engineers on the abundance of habitat-providing species adds additional understanding of how biological interactions can drive spatial distribution of foundation species.

Numerous abiotic gradients that can influence coral growth and survival exist across the fringing reef and mid-lagoon habitats around Moorea. As the habitat immediately adjacent to the shoreline, the fringing reef is subjected to increased terrestrial runoff that can increase sedimentation, turbidity, nutrient pollution, freshwater intrusion, and rapid and extreme fluctuations in temperature and salinity^5,55,56^. All these stressors can independently and in some cases synergistically reduce coral growth rates and promote coral mortality^1,57,58^. In our field experiment, we found that when protected from fishes, *A. pulchra* grew faster and exhibited partial mortality from non-consumptive causes far less in the mid-lagoon compared to the fringing reef. Although our snapshot of abiotic conditions at experimental sites was unable to detect differences in abiotic conditions between the two habitats, our findings for *A. pulchra* suggest that from the perspective of coral physiology, conditions in the mid-lagoon provide a superior habitat for this species compared to the fringing reef habitat.

One plausible explanation for the difference in TLE and related mass change of corals in the mid-lagoon compared to the fringing reef is breakage via hydrodynamic forces; higher wave energy in the mid-lagoon compared to the fringing reef could cause breakage, reducing TLE and coral growth rates. However, when we compared changes in TLE for caged vs. exposed corals in the mid-lagoon, which were subjected to the same hydrodynamic forces, we found that the TLE of caged corals increased on average 7 cm more than those exposed to predators. These results do not support wave energy as a mechanism reducing growth rates of exposed corals in the mid-lagoon. Alternatively, because our experiment was conducted during the austral winter, a period during which wave energy is low compared to during the austral summer^5^, it is plausible that physical wave energy at some point during the year is too strong for *A. pulchra* to persist in the mid-lagoon. However, a year-long caging experiment at a site ~ 300 m from shore found that *A. pulchra* could both thrive and remain anchored at this distance from shore^47^. Moreover, our island-wide surveys found *A. pulchra* within 110 m of the reef crest, indicating that this species can persist relatively close to this high wave energy habitat. Thus, it does not appear that hydrodynamic forcing precludes *A. pulchra* from existing in the mid-lagoon, and even in areas relatively close to the reef crest.

Nitrogen (N) loading on coral reefs, particularly anthropogenically-derived N in the form of nitrate, can impair coral growth^8^, increase the incidence and severity of disease^3,9^, promote the proliferation of algal competitors, and compromise the ability for corals to mount an immune response^59^. However, perhaps one of the most important effects of nutrient pollution is its interaction with temperature stress to make corals more susceptible to bleaching^60^. Indeed, our surveys found that both the presence and the amount of *A. pulchra* were inversely related to % N, lending support to the hypothesis that nutrients and temperature interact to shape the distribution of *A. pulchra* around Moorea. This finding is particularly relevant as Moorea experienced a severe thermal stress event in 2019^61^, causing the mass mortality of corals on the forereef and likely also in the lagoon habitat where this study was conducted. Nitrogen availability in the waters surrounding Moorea varies seasonally and across habitats but is typically highest in the fringing reef^5,13^. Moreover, nutrient enrichment in Moorea tends to be associated with terrestrial runoff and thus is likely correlated with a higher abundance of additional inputs that could harm corals like sediments, toxins, and pathogens^5,14^. Some of these nearshore waters chronically experiencing N loading have undergone shifts from coral- to algal-dominated states over the past decade, compromising conditions for coral success^5^. Thus, sublethal effects of nutrient pollution could impact *A. pulchra* through numerous mechanisms and contribute to our observed patterns of *A. pulchra* performance and distribution.

In our system, predation can exert a strong influence on *A. pulchra* over short timescales and appears to be a fundamentally important mechanism influencing the success of juvenile-sized corals and thus may influence the establishment of new *A. pulchra* thickets. Contrasting with predation, abiotic drivers such as N enrichment, which were negatively associated with the presence of staghorn thickets, likely operate on longer or episodic timescales to shape the habitat distribution of *A. pulchra*. These findings underscore the nuanced interplay between environmental and biotic factors that dictate the local distribution of a foundation species within a seascape. The response of foundation species to growth–predation risk tradeoffs can shape the distribution of key habitat forming species, with profound consequences for community structure, diversity, and ecosystem functions. Understanding these relationships and the responses of foundation species in the face of rapidly changing conditions is essential to predict ecosystem responses to local and global stressors to inform management actions and guide conservation efforts aimed at increasing the resilience of complex ecosystems like coral reefs.

## Methods

### Study species

*Acropora pulchra* is a branching coral species and an important creator of habitat in shallow lagoons throughout the Pacific. This foundation species can form large monospecific stands (i.e., thickets) that provide habitat for at least 85 species of fishes from 25 different families at our study site^32^. The thickets are frequently colonized by territorial algal-farming damselfish that fiercely protect them from coral predators^32,51–53^. *A. pulchra* was historically abundant throughout the fringing reef and mid-lagoon habitats of Moorea^31^, but its spatial distribution has been more limited in recent decades due largely to limited recovery after major disturbance events several decades ago^62^.

### Island-wide Spatial patterns of distribution of *A. pulchra*

We conducted surveys of the coral reefs surrounding the island of Moorea (17º 30’ S, 149º 50’ W). Moorea is a high volcanic island in the central south Pacific 20 km west of Tahiti and has a barrier reef 1 to 1.5 km from shore that encircles Moorea and forms a system of narrow, shallow (~ 2 to 15 m deep) lagoons. To examine the distribution of *A. pulchra* in the lagoons around the island, we surveyed 188 sites around Moorea between January and May 2016. Sites were at least 0.5 km apart, were distributed around the entire island, (Fig. 1a) and were spaced to maximize coverage of the different reef habitats within the lagoons, including the fringing reefs, mid-lagoon, back reefs, reef passes, and bays^5^. Sites ranged in distance from shore from 30 to 1257 m. At each site two snorkelers conducted 10-minute swims in opposite directions, covering an area of up to ~ 5000 m^2^ and recorded all observed colonies of *A. pulchra* ≥ 1 m diameter. In addition, in May 2016 a subset of 169 of the 188 sites were surveyed. All colonies encountered that were at least 10 cm in diameter were recorded and their sizes estimated (maximum diameter and perpendicular to this measure; L x W) to the nearest 10 cm.

In addition to the surveys of *A*. *pulchra*, island-wide patterns of nutrient (N) enrichment were quantified. To do this, we collected samples of *Turbinaria ornata* (henceforth *Turbinaria*) from 168 of the 188 sites surveyed for the presence or absence of *A. pulchra*. Like other macroalgae, *Turbinaria* responds to N pulses by storing surplus N^63^, and, consequently, N tissue content is believed to be an excellent time-integrated indicator of N availability^64–67^. Sampling was conducted over ~ 3 weeks in May 2016. At each of the sites, we collected thalli from 10 different patches of *Turbinaria* across an area of ~ 500 m^2^. Samples were immediately placed in a cooler and transported to the laboratory. One blade from each of 10 thalli was sampled at 5 cm below the apical tip. Blades were scrubbed of epiphytes and rinsed with fresh water before being dried at 60° C to a constant mass and ground to a fine powder. Total N content was determined via elemental analysis using a CHN CarloErba elemental analyzer (NA1500) at the University of Georgia, Center for Applied Isotope Studies.

Several different statistical analyses of island-wide spatial patterns were done to explore relationships between nutrient enrichment, site location (distance from shore, side of the island), and the presence-absence and abundance of *A*. *pulchra*. First, to assess if the probability of *A. pulchra* thickets being present at sites surveyed around the island was related to N availability (%N in *Turbinaria*) and distance from shoreline, we used a generalized linear model (GLM) with a binomial distribution and a logit link function. For this model, %N and distance from shoreline (m) were interacting fixed factors, and island shore was a fixed, non-interacting factor. We only included thickets, i.e., colonies ≥ 1 m diameter, for these analyses as their larger size suggest that they have been established in these locations to reach this size and did not recently recruit. We also tested for a correlation between %N and distance to shore using a Pearson correlation test. Second, to explore patterns of abundance, we estimated the total area of all *A. pulchra* colonies observed in our surveys by calculating the area of an ellipse using the values of the two diameters measured in the field to obtain radius measures. These estimates were then summed to quantify the amount of live *A. pulchra* observed at each site and habitat. We investigated the relationships between the abundance (area of *A. pulchra* present) at each site and distance to shore, % N, and island shore via GLM. For this GLM, based on results from our presence – absence analyses above, we considered distance to shore, % N, and island shore fixed non-interacting factors. Area measures were log-transformed to meet model assumptions.

### Field experiment to assess habitat-specific patterns of growth and mortality of *A. pulchra*

In the austral winter of 2017, we conducted a field experiment to assess spatial patterns of growth and mortality of *A. pulchra* in two lagoon habitats (mid-lagoon, fringing reef) and to explore potential underlying mechanisms. Twenty sites on the north shore of the island were selected (10 in the mid-lagoon, 10 on the fringing reef); these were a subset of the larger group of 188 survey sites (Fig. 1e). Sites were selected to span most of the north shore and to represent a gradient of N availability^5,6^. The mid-lagoon habitat, located offshore of deep-water channels, consists of a sandy bottom in 1 to 3 m of water with a matrix of both live and dead coral. Colonies of massive *Porites* spp. (henceforth called bommies) were numerous and ranged in size from < 10 cm diameter to > 4 m diameter. Live *Porites* bommies provide habitat for a diversity of fishes and other reef-dwelling inhabitants^67^, while dead bommies constitute important substrate for the recruitment and growth of branching corals (e.g., *Pocillopora* and *Acropora* spp.) that similarly provide important habitat for fishes^68^. They also provide attachment space for macroalgae (e.g., *Turbinaria ornata*, *Sargassum pacificum*) and a range of invertebrates.

The fringing reef habitat is directly adjacent to shoreline, often with a shallow shelf (< 1 to 2 m depth) extending outwards that steeply slopes down to meet relatively deep water (> 10 m) in some locations. Benthic communities on the fringing reef vary widely but include a mosaic of sand, coral rubble, live coral, hardbottom, and dead coral heads colonized by turf algae and macroalgae.

We surveyed the 20 experimental sites to measure key biotic and abiotic attributes that may influence coral growth and predation. At each site, we quantified the abundance and biomass of fishes via GPS enabled surveys. A snorkeler swam for up to 30 min at a speed of roughly 5 to 10 m per minute while counting all fishes *≥* 10 cm in length in a 5 m wide swath while towing a float with a handheld GPS programmed to take a waypoint every 15 s. This allowed us to calculate the total area of the reef surveyed to convert counts to densities (see^69^. Fish lengths were converted to biomass estimates using published length-weight relationships^70^. All sites were surveyed two times on different days and the mean biomass density of corallivorous fishes was calculated for each site (see Table S1 for list of corallivorous species observed in the surveys). Nutrient enrichment was quantified via the % N tissue content of *Turbinaria* as described above; *Turbinaria* samples were taken in August 2017, midway through the experimental period.

Sedimentation rates were measured at each site using sediment traps constructed from lightweight plastic containers (17.5 cm x 12.5 cm x 2.5 cm) fitted with astro turf secured with cable ties. Each trap was mounted on a rack made of PVC-coated galvanized wire mesh, which was affixed to open substrate on the tops of patch reefs, avoiding areas with coral or macroalgae. Five replicate traps were deployed at each site, with racks placed at least 5 m apart. Traps were left in place for 7 days, after which they were carefully collected and transported to the lab in individual sealed plastic bags. In the lab, samples were allowed to settle for 24 h, excess water was siphoned off, and the samples were frozen for transport to the University of California, Santa Barbara for further analysis. Frozen samples were thawed at room temperature in individual petri dishes. Once thawed, samples were placed in a drying oven and dried at 60 °C until they reached a constant weight, typically 48 h. Final dry weights for each sample were obtained by weighing the dried samples on an analytical grade balance to the nearest 0.001 g.

To quantify coral growth and corallivory, we deployed five pairs of caged and uncaged nubbins (i.e., ~ 6 cm long, finger-like fragments) of *A. pulchra* (*n* = 20 corals per site; *n* = 400 corals total). Nubbins were attached to experimental platforms that served as experimental replicates. Each platform was constructed of a 15 × 15 cm square of stainless-steel mesh that had four polypropylene 50 mL Falcon^®^ centrifuge tube caps attached via a small bolt inserted through a hole drilled in the cap and secured onto the platform with a washer and a nut. To exclude fish corallivores, a 7.5 × 15 × 15 cm (width x length x height) cage constructed of PVC-coated mesh (2.54 × 2.54 cm hole size) was attached to one side of the platform via cable ties such that it enclosed two of the four centrifuge caps (Figure S1). At each site we secured five 30 cm stainless steel threaded rods into pre-drilled holes ~ 10 cm deep drilled into dead bommies or hard substrate. A single experimental platform was secured to each threaded rod via a set of nuts and washers, and elevated ~ 10 cm from the substrate. All experimental platforms were located in water 1–3 m in depth. When selecting locations to deploy experimental platforms, care was taken to select areas that were not inside of damselfish territories.

On each experimental platform we transplanted two exposed and two caged *A*. *pulchra* nubbins (*n* = 10 per treatment at each site). All corals were collected over a period of 2 days from the same large thicket of *A. pulchra* at a single lagoon site on the northwest shore of the island (Fig. 1e), and thus it was assumed that all colonies came from the same putative genotype. Corals were collected the day before deployment using bone cutters to separate a single branch tip (henceforth referred to as a “coral”) from the colony. Each coral was placed into an individual resealable bag filled with seawater, placed into a cooler, and transported to flow-through seawater tables in the laboratory. On the same day of collection, each coral nubbin was secured in a 3 cm section of a 50 mL centrifuge tube using a two-part marine epoxy. Each coral was individually numbered using underwater paper placed inside the centrifuge tube. We estimated coral growth using two metrics: total linear extension (cm TLE; i.e., the sum of all branches) and buoyant weight (grams)^71^. On the day of deployment, the mean (± SE) height of nubbins was 5.83 cm (± 0.05) and the mean mass was 10.76 g (± 0.10). On the day following collection, coral nubbins were randomly assigned to treatment and transplanted to experimental platforms in a random, pre-determined design. Experimental corals were checked every 5 to 7 d to ensure that platforms were intact and cages remained closed and to record observations of corallivory, paling, or any other changes in the corals. At the conclusion of the experiment (October 1 and 3, 83 d after initiation) we photographed all corals in situ, visually estimated partial mortality (i.e., skeletal area with no live tissue), and measured them for buoyant weight and total linear extension in the laboratory to quantify changes in skeletal mass, height, and total linear extension.

As detailed below, our spatial surveys of abundance of *A*. *pulchra* revealed much higher abundances on the fringing reef than in the mid-lagoon. Based on this finding, we posited and tested two main hypotheses about spatial variation in demographic performance (growth, mortality) of *A*. *pulchra* to explore possible mechanisms that account for the observed pattern. Our first supposition was that abiotic conditions promote faster growth of *A. pulchra* at nearshore habitats (i.e., fringing reefs) compared to offshore (i.e., mid-lagoon) habitats. Our second postulate was that *A. pulchra* performs worse when exposed to corallivorous fishes in the mid-lagoon compared to the fringing reef due to greater rates of consumption.

To test the first hypothesis, we assessed differences in percent change in mass and partial mortality at the end of the experiment for caged corals only (i.e., in the absence of predation) transplanted to the fringing reef and mid-lagoon. We used a Generalized Linear Mixed Model (GLMM) that used a Gaussian family with an identity link function. This model considered habitat a fixed effect and included a random intercept to account for potential variability among sites. Percent change in mass data were square root transformed to meet model assumptions. To evaluate the effects of habitat on partial coral mortality, we employed a two-part hurdle model. This approach is appropriate for modelling zero-inflated proportional data where a large number of observations are zeros and the remaining values are bounded between 0 and 1. First, we modeled the binary component of the mortality data using a GLMM with a binomial distribution with a logit-link. This model included habitat as a fixed effect and site and a random intercept to account for potential non-independence of observations within sites. We then subset the data to include only observations with nonzero proportional mortality. To ensure these data conformed to the assumptions of the beta distribution, values were rescaled to fall between 0 and 1. We modeled these data using a GLMM with a beta distribution with a logit link and included habitat as a fixed effect and site and a random intercept.

To test our second hypothesis that coral growth was worse in the mid-lagoon compared to the fringing reef when exposed to corallivorous fishes, we assessed differences in the percent change in mass and total linear extension for uncaged (i.e., exposed to predation) corals only. To investigate if hydrodynamic forces may have contributed to observed patterns in growth or linear extension in the mid-lagoon, we compared changes in total linear extension between caged and uncaged corals transplanted to the mid-lagoon. For these analyses, we used GLMMs as described above for percent change in mass. Total linear extension data used to compare total linear extension in caged vs. uncaged transplants in the mid-lagoon were square root transformed. However, even with this transformation these data failed Levene’s Test for homogeneity of variance and thus should be interpreted accordingly. Cohen’s d effect sizes, which provide a quantitative measure of the difference between two groups, were calculated for growth, partial mortality, and TLE comparisons. Generally, Cohen’s d effect sizes are considered small, medium, and large when Cohen’s d is equal to 0.2, 0.5, and 0.8, respectively.

We quantified the tradeoff between growth and predation by subtracting the mean growth rate (measured as percent change in mass) of caged corals on a platform (*n* = 2) from the mean growth rate of corals exposed to predators (*n* = 2) on the same platform. Thus, a value of zero indicates that corallivores had no effect on growth at site, as corals exposed to predators grew as much as corals protected from predators. Conversely, negative numbers indicate a predation effect, as corals exposed to predators grew less than corals protected from predators inside of cages. We then used a linear model to assess the relationship between these calculated values and mean corallivorous fish biomass across all experimental sites.

We conducted one-factor ANOVAs to investigate if corallivorous fish biomass, nutrient levels (%N content of *Turbinaria*) or sedimentation (dry sediment weight, g) differed between mid-lagoon and fringing reef habitats. For these models, mean values were calculated for each site and used as an individual replicate (*n* = 10 per habitat). Because we were interested in the effects of the abiotic factors %N and sedimentation on coral growth in the absence of predation, for these analyses we excluded corals that were exposed to coral predators and calculated the mean percent change in mass of caged corals at each site. We then performed individual linear regressions that regressed coral growth (measured as percent change in mass over the course of the experiment) against %N of *Turbinaria* at each site or dry sediment weight (g) collected from the sedimentation traps deployed during the experiment. Partial mortality was log-transformed for these analyses.

We used PERMANOVA to test for differences in corallivorous fish community composition between habitats. For these analyses, we combined the two surveys conducted at each site and separately assessed the abundance and biomass of corallivorous fish species observed during our surveys. If the PERMANOVA detected a significant difference in community composition, we then used ANOSIM to explore species contributions to dissimilarity between habitats. We visualized differences in the abundance and biomass of corallivorous fish communities via non-metric multidimensional scaling (NMDS) using a random starting configuration and Bray-Curtis distance based on 9999 permutations.

All analyses were conducted in R version 4.3.2^72^ with general data manipulations conducted with tidyverse^73^. ANOSIM, PERMANOVA, and NMDS analyses were conducted using the vegan package in R^74^. Mixed effects and GLMM models were run with the glmmTMB package^75^ and model fit and residual assumptions were evaluated using the DHARMa package^76^.

## Supplementary Information

Below is the link to the electronic supplementary material.

Supplementary Material 1

## Data Availability

All data sets and code utilized for this research are publicly available from the Environmental Data Initiative (EDI) Data Portal: https://doi.org/10.6073/pasta/61c73b28108cfaf6e2c130b339a3446f.
