# Supplementary material for: Growth-predation risk tradeoffs constrain the local distribution of a thicket-forming Staghorn coral to marginal reef habitats
Source: Sci Rep. 2025 Oct 23;15:37119. doi: 10.1038/s41598-025-21028-z (PMC12549834; doi:10.1038/s41598-025-21028-z)
Supplement: Supplementary file 1 — Supplementary Material 1 [file 41598_2025_21028_MOESM1_ESM.docx]

**Supplemental Material**

**
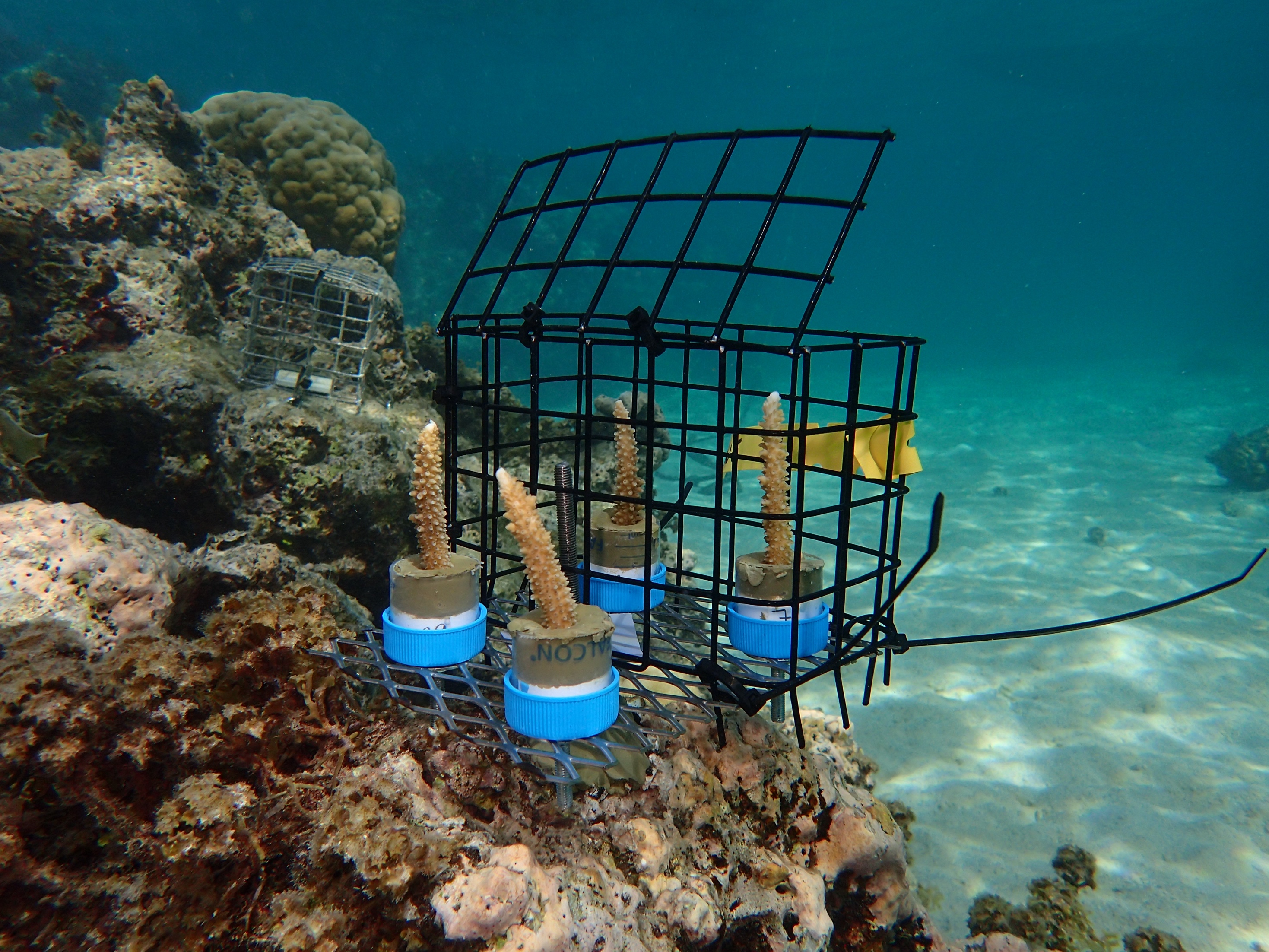
**

**Figure S1.** Photograph of a single replicate with experimental *Acropora pulchra* corals exposed to predators (foreground) and protected from predators by cages (background). Photo credit: M. Ladd.

**Figure S2**. Box-and-whisker plots of (a) corallivorous fish biomass, (b) % N tissue content, and (c) sediment dry weight (g) for the two habitats included in this study (n = 10 sites per habitat). Statistics from one-way ANOVA. Mean coral performance (% change in mass of exposed corals - % change in mass of caged corals) at each site regressed against (d) the mean biomass (g m^-1^) of corallivores. Percent change in mass over the duration of the experiment for caged corals only regressed against (e) mean dry sediment weight (f), and percent nitrogen tissue content of *Turbinaria ornata* samples taken at each site in August 2017. Statistics from linear regression.

**Figure S3.** Non-metric multidimensional scaling (NMDS) ordination of fish community composition at fringing reef and mid-lagoon habitats based on Bray-Curtis dissimilarity of species biomass (**a**) and abundances (**b**). Vectors represent species that were significantly correlated with the ordination axes (p < 0.05); only species with significant correlations are shown. Statistics from PERMANOVA.

**Table S1**: List of fish species observed during fish surveys and their classification as corallivores and skeleton removers. Fish were only included in mean corallivore biomass calculations if they were classified as Corallivore = X. For parrotfishes (Family Scaridae), only fish ≥20 cm TL were included in corallivore biomass calculations.

| **Species** | **Family** | **Corallivore** | **Skeleton Remover** |
| --- | --- | --- | --- |
| *Acanthurus guttatus* | Acanthuridae |  |  |
| *Acanthurus lineatus* | Acanthuridae |  |  |
| *Acanthurus nigricans* | Acanthuridae |  |  |
| *Acanthurus nigricauda* | Acanthuridae |  |  |
| *Acanthurus nigrofuscus* | Acanthuridae |  |  |
| *Acanthurus nigroris* | Acanthuridae |  |  |
| *Acanthurus pyroferus* | Acanthuridae |  |  |
| *Acanthurus triostegus* | Acanthuridae |  |  |
| *Arothron meleagris* | Tetraodontidae | X | X |
| *Balistapus undulatus* | Balistidae | X | X |
| *Carcharhinus melanopterus* | Chondrichthyes |  |  |
| *Chaetodon auriga* | Chaetodontidae | X |  |
| *Chaetodon citrinellus* | Chaetodontidae | X |  |
| *Chaetodon ephippium* | Chaetodontidae | X |  |
| *Chaetodon lunula* | Chaetodontidae | X |  |
| *Chaetodon lunulatus* | Chaetodontidae | X |  |
| *Chaetodon ornatissimus* | Chaetodontidae | X |  |
| *Chaetodon reticulatus* | Chaetodontidae | X |  |
| *Chaetodon trifascialis* | Chaetodontidae | X |  |
| *Chaetodon ulietensis* | Chaetodontidae | X |  |
| *Chaetodon vagabundus* | Chaetodontidae | X |  |
| *Chlorurus spilurus* | Scaridae | X | X |
| *Ctenochaetus striatus* | Acanthuridae |  |  |
| *Diodon hystrix* | Diodontidae |  |  |
| *Forcipiger longirostris* | Chaetodontidae |  |  |
| *Heniochus chrysostomus* | Chaetodontidae | X |  |
| *Leptoscarus vaigiensis* | Scaridae |  |  |
| *Melichthys vidua* | Balistidae |  |  |
| *Naso lituratus* | Acanthuridae |  |  |
| *Naso unicornis* | Acanthuridae |  |  |
| *Pygoplites diacanthus* | Pomacanthidae |  |  |
| *Rhinecanthus aculeatus* | Balistidae | X | X |
| *Scarus altipinnis* | Scaridae | X | X |
| *Scarus globiceps* | Scaridae | X | X |
| *Scarus oviceps* | Scaridae | X | X |
| *Scarus psittacus* | Scaridae | X | X |
| *Siganus spinus* | Siganidae |  |  |
| *Sufflamen bursa* | Balistidae |  |  |
| *Zebrasoma scopas* | Acanthuridae |  |  |
| *Zebrasoma veliferum* | Acanthuridae |  |  |

**Table S2**: Results of SIMPER analysis identifying corallivorous fish species contributing most to community dissimilarity in biomass between fringing reef and mid-lagoon habitats. Species are ordered by their contribution to average Bray–Curtis dissimilarity. Only species up to 95% cumulative dissimilarity are included.

| **Species** | **Contribution to Community Dissimilarity** | **Cumulative Contribution to Dissimilarity** |
| --- | --- | --- |
| *Chlorurus spilurus* | 38.6% | 38.6% |
| *Scarus psittacus* | 32.5% | 71.1% |
| *Scaurs oviceps* | 9.8% | 80.9% |
| *Balistapus undulatus* | 5.2% | 86.1% |
| *Scarus altipinnis* | 4.6% | 90.7% |
| *Scarus globiceps* | 2.7% | 93.4% |
| *Chaetodon lunula* | 1.6% | 95.0% |
